# The Association of *APOE* Genotype with Cognitive Function in Persons Aged 35 Years or Older

**DOI:** 10.1371/journal.pone.0027415

**Published:** 2011-11-14

**Authors:** Gerbrand J. Izaks, Ron T. Gansevoort, Aafke M. van der Knaap, Gerjan Navis, Robin P. F. Dullaart, Joris P. J. Slaets

**Affiliations:** 1 University Center for Geriatric Medicine, University Medical Center Groningen, University of Groningen, Groningen, The Netherlands; 2 Alzheimer Center Groningen, University Medical Center Groningen, University of Groningen, Groningen, The Netherlands; 3 Department of Nephrology, University Medical Center Groningen, University of Groningen, Groningen, The Netherlands; 4 Department of Endocrinology, University Medical Center Groningen, University of Groningen, Groningen, The Netherlands; Georgetown University Medical Center, United States of America

## Abstract

*APOE* genotype is associated with the risk of Alzheimer's disease. In the present study, we investigated whether *APOE* genotype was associated with cognitive function in predominantly middle-aged persons. In a population-based cohort of 4,135 persons aged 35 to 82 years (mean age (SD), 55 (12) years), cognitive function was measured with the Ruff Figural Fluency Test (RFFT; worst score, 0 points; best score, 175 points). *APOE* genotype (rs429358 and rs7412) was determined by polymerase chain reaction. The mean RFFT score (SD) of the total cohort was 69 (26) points. Unadjusted, the mean RFFT score in homozygous *APOE* ε4 carriers was 4.66 points lower than in noncarriers (95% confidence interval, -9.84 to 0.51; *p* = 0.08). After adjustment for age and other risk factors, the mean RFFT score in homozygous *APOE* ε4 carriers was 5.24 points lower than in noncarriers (95% confidence interval, -9.41 to -1.07; *p* = 0.01). The difference in RFFT score was not dependent on age. There was no difference in RFFT score between heterozygous *APOE* ε4 carriers and noncarriers. The results indicated that homozygous *APOE* ε4 carriers aged 35 years or older had worse cognitive function than heterozygous carriers and noncarriers.

## Introduction

It is well established that the risk of Alzheimer's disease is increased in *APOE* ε4 carriers [1]. Probably, this is caused by diverse pathogenic mechanisms as apolipoprotein E (ApoE) plays a role in various processes in the brain including the production and clearance of amyloid-β, and the maintenance and repair of synapses and neurons [2]. As some of these processes occur at all ages, it is likely that ApoE also contributes to structural and functional changes in the brain of young persons. Therefore, *APOE* genotype may be related to cognitive function many years before cognitive impairment becomes clinically apparent.

Only a few studies on the relationship of *APOE* genotype with cognitive function included young adults. Three studies were large enough to examine the association between *APOE* genotype and cognitive function in successive groups of age [3]–[6]. One study showed no association of *APOE* genotype with cognitive function in young adults [3], [6], but two other studies found that *APOE* ε4 carriers aged 40 years or older had worse cognitive function than noncarriers of similar age. However, the latter studies were performed in strongly selected populations with a relatively high frequency of the *APOE* ε4 allele [4], [5]. More importantly, one of the studies essentially included one extended family [4].

The aim of this study was to investigate the association of *APOE* genotype with cognitive function in both young and older persons from the general population. The study included 4,135 persons who were aged 35 to 82 years.

## Materials and Methods

### Study population

The study population comprised all participants of the third survey of the Prevention of Renal and Vascular ENd-stage Disease study (PREVEND) that is being conducted in the city of Groningen, the Netherlands. The PREVEND study was designed to investigate prospectively the natural course of (micro)albuminuria and its relation to renal and cardiovascular disease in the general population. Details of the study protocol have been published elsewhere [7], [8], and can be found at www.iadb.nlwww. prevend.org. The total cohort included 8,592 persons at baseline (1997–1998). Of these, 8,574 persons completed the first survey and were followed over time. The second survey was performed from 2001 to 2003 (N = 6,894) and the third survey was performed from 2003 to 2006 (N = 5,862); thus, compared to the cohort at baseline, the participation rate was 80% and 68%, respectively.

### Cognitive function

Cognitive function was measured with the Ruff Figural Fluency Test (RFFT)[9], [10]. The test was added to the measurements of PREVEND in the third survey. The RFFT is a measure of nonverbal fluency. Fluency is generally seen as an executive function and is sensitive to early cognitive dysfunction [11]. The RFFT has five parts and requires respondents to draw as many different designs as possible within one minute per part by connecting patterns of dots [10]. The RFFT is sensitive to changes in executive function in both young and older persons [9], [12]. The main outcome measure of the RFFT is the total number of unique designs, which varies from 0 points (worst score) to 175 points (best score). In the PREVEND study, each RFFT was analyzed by two trained and independent examiners (intraclass correlation coefficient, 1.00; 95%CI, 0.99 to 1.00)[12].

All 5,862 participants of the third survey of the PREVEND study were required to perform the RFFT, and the test was completed by 4,158 persons (71%). The RFFT scores of the other 1,704 persons (29%) were not known: 1,271 persons (22%) refused to perform the RFFT, and 434 persons (7%) had missing RFFT scores for other reasons.

### Age and educational level

Age was defined as the age in full years on the date of performance on the RFFT. Educational level was divided into four groups according to the International Standard Classification of Education (ISCED) [13]: primary school, ISCED 0 to 1 (0 to 8 years of education); lower secondary education, ISCED 2 (9 to 12 years of education); higher secondary education, ISCED 3–4 (13 to 15 years of education); university, ISCED 5 (16 or more years of education).

### *APOE* genotyping

Genotyping was performed at the University Medical Center Groningen and at the Laboratory of Experimental Vascular Medicine, Academic Medical Center Amsterdam, the Netherlands. DNA was extracted from whole blood using the Qiamp mini kit (Qiagen). *APOE* genotypes (rs429358 and rs7412) were determined by allelic discrimination on a TaqMan 7500 Real Time PCR system, using the single-nucleotide polymorphism (SNP) genotyping mixes C-3084793-20 and C-904973-10 and Taqman Universal PCR mastermix No AmpErase (Applied Biosystems, Nieuwerkerk a/d IJssel, the Netherlands). The method has been validated against a previously described restriction isotyping procedure [14], [15]. Assays were carried out according to the manufacturer's recommendations on an ABI 7900HT apparatus.

### Other measurements

Cardiovascular risk factors were measured because they are important non-genetic risk factors for Alzheimer's disease and cognitive decline [16]. Diabetes mellitus was defined as either a fasting glucose equal to or greater than 7 mmol/L, or a non-fasting glucose equal to or greater than 11.1 mmol/L, or the use of glucose lowering drugs [17]. Smoking was defined as current smoking or cessation of smoking less than one year before the study. Body Mass Index (BMI) was calculated as weight in kilograms divided by height in meters squared. Blood pressure was automatically measured in supine position during ten minutes on both visits. Blood pressure values are given as the mean of the last two recordings of both visits. Plasma glucose and total cholesterol were measured by dry chemistry (Eastman Kodak, Rochester, NY). High density lipoprotein (HDL) cholesterol was measured with a homogeneous method (direct HDL, Aeroset TM System, Abbott Laboratories, Abbott Park, IL). Non-HDL cholesterol was calculated as total cholesterol minus HDL cholesterol. Albuminuria was determined in two consecutive 24-hour urine samples by nephelometry. Elevated albuminuria was defined as albuminuria equal to or greater than 30 mg/day. A history of cardiovascular and cerebrovascular events was defined as a prior cardiac or cerebrovascular event for which subjects had been hospitalised. Data on disease history were derived from a questionnaire at baseline and during follow-up obtained from the Dutch national registries of hospital discharge diagnoses and death certificates. Data on drug use were obtained from the InterAction DataBase (IADB; www.iadb.nl) of the departments of PharmacoEpidemiology and PharmacoEconomics of the Groningen Graduate School of Medical Sciences which comprises pharmacy-dispending data of the community-pharmacists in the study region of the PREVEND study [18].

### Ethics statement

The PREVEND study has been approved by the Medical Ethical Committee (METc) of the University Medical Center Groningen, Groningen, the Netherlands, and is conducted in accordance with the guidelines of the Declaration of Helsinki. Written informed consent was obtained from all participants.

### Statistical analysis

Differences in numerical variables were tested with the two-tailed independent-samples t test or, if appropriate, one-way analysis of variance. Differences in categorical variables were tested by χ^2^ test or, if appropriate, Fisher exact test. The association between RFFT score and *APOE* genotype was analyzed by multiple linear regression analysis. *APOE* genotype was entered in the regression model as two dummy variables: *APOE* ε4 heterozygosity (no/yes) and *APOE* ε4 homozygosity (no/yes). By definition, the noncarrier genotype was the reference category. The regression model also included the variables age, gender, educational level, diabetes mellitus, smoking status, BMI, systolic blood pressure, glucose, HDL cholesterol, non-HDL cholesterol, and elevated albuminuria. Interaction between *APOE* genotype and age was tested by entering the product terms *APOE* ε4 heterozygosity x Age and *APOE* ε4 homozygosity x Age in the regression model. In all regression models, the variables age, BMI, systolic blood pressure, glucose, HDL cholesterol and non-HDL cholesterol were entered as continuous variables. All other variables were entered as categorical variables. The level of statistical significance was set at 0.05. Statistical analysis was done with SPSS 16.0 for Windows (SPSS Inc., Chicago, IL).

### Additional analyses

Due to its design, the total PREVEND cohort comprised a relatively high number of persons with elevated albuminuria. Because this may influence some data analyses, all analyses were repeated in a representative sample of the Groningen population. This so-called Groningen Random Sample is a subset of the total PREVEND cohort, and was created by stratified random sampling with strata based on albuminuria [7]. The prevalence of albuminuria in the Groningen Random Sample is equal to the prevalence in the general population. At the third survey, the Groningen Random Sample included 2,404 persons.

Secondly, the analyses were repeated after exclusion of 172 persons (4%) of non-European descent. This group mainly included persons of Asian descent but also 30 persons of African descent (Table S1). Concordant with studies in populations of African descent, the prevalence of the *APOE* ε4 allele was relatively high in this group (Table S2).

Thirdly, the analyses were repeated after exclusion of *APOE* ε2/ε4 carriers because the *APOE* ε2 allele is associated with protection against Alzheimer's disease [1], and the effect of the *APOE* ε2/ε4 genotype on cognitive function is unclear. The total study population included 605 *APOE* ε2 carriers (15%): *APOE* ε2/ε2 carriers, 30 (1%); ε2/ε3 carriers, 483 (12%); ε2/ε4 carriers, 92 (2%).

Finally, the analyses were repeated after exclusion of all *APOE* ε2 carriers.

## Results

The RFFT was completed by 4,158 persons. Twenty persons (0.5%) who completed the RFFT were excluded because their educational level was not known and three persons (0.1%) were excluded because their age was less than 35 years. Thus, the total study population comprised 4,135 persons (Table 1).

**Table 1:** Characteristics of the study population.

N (%)	4135	(100)
**Gender, N (%)**		
Women	1978	(48)
Men	2157	(52)
**Ethnicity**		
European	3963	(96)
Other	172	(4)
**Age, mean (SD), y**	55	(12)
**Age, N (%), y**		
35–44	920	(22)
45–54	1277	(31)
55–64	1002	(24)
65–74	691	(17)
≥75	245	(6)
**Educational level, N (%)**		
Primary school	406	(10)
Lower secondary education	1225	(29)
Higher secondary education	1108	(27)
University	1396	(34)
***APOE*** ** ε4 genotype, N (%)**		
All	3855	(93)
Homozygous carrier (ε4/ε4)	101	(2)
Heterozygous carrier (ε2/ε4 or ε3/ε4)	1065	(26)
Noncarrier	2689	(65)
**RFFT score, mean (SD)**	69	(26)

The *APOE* genotype could be determined in 93% of the total study population (Table 1). The frequency of the various *APOE* genotypes was comparable to their frequency in other Dutch populations [19], [20]. Twenty-eight percent of the study population was *APOE* ε4 carrier of whom 2% was *APOE* ε4 homozygous and 26% was *APOE* ε4 heterozygous (Table 1). There was no difference in genotype frequency between persons who performed the RFFT and persons who did not perform the RFFT (data not shown).

### *APOE* genotype and cognitive function

**Figure 1:**
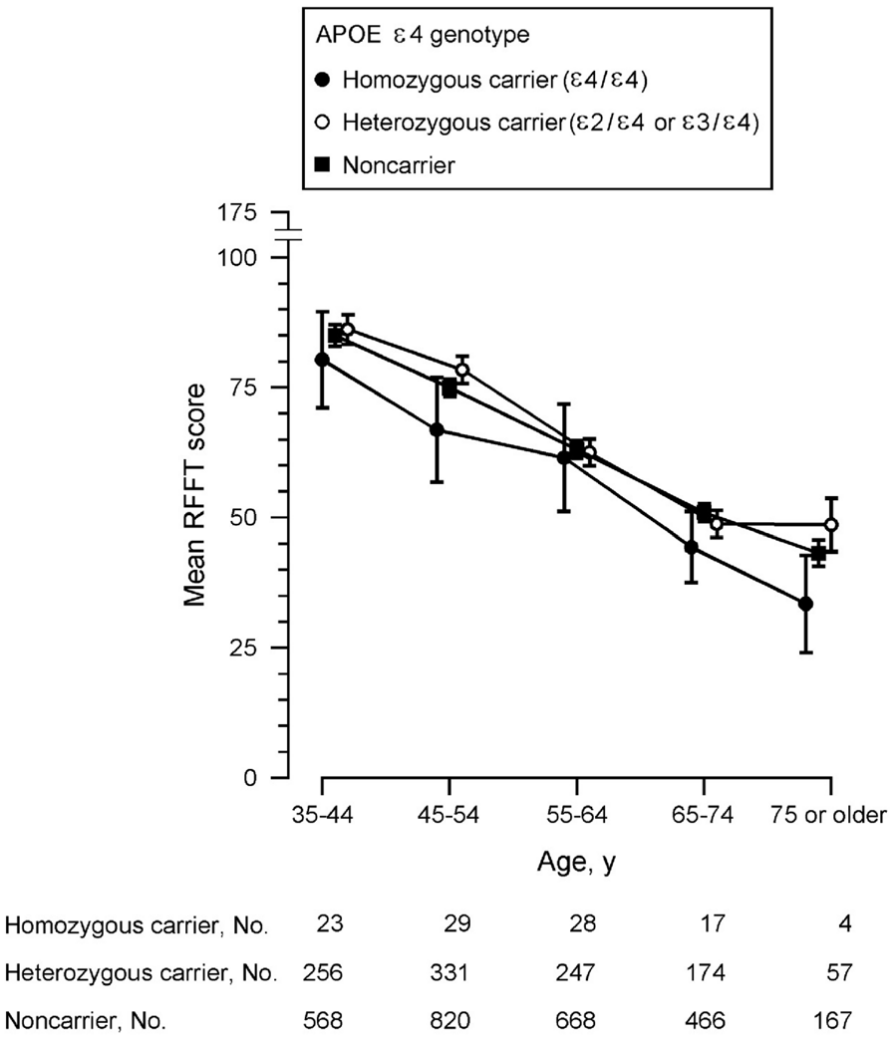
RFFT score dependent on *APOE* ε4 genotype and age. For clarity, data are presented as mean and 95% confidence interval of ten-year age groups.

RFFT scores were normally distributed and homozygous *APOE* ε4 carriers had lower RFFT scores than noncarriers. This difference was observed across all ages (Figure 1). Overall, the mean difference in RFFT score between homozygous *APOE* ε4 carriers and noncarriers was 4.66 points (95% confidence interval, -9.84 to 0.51; *p* = 0.08). This could not be ascribed to differences in demographic characteristics as there were no statistically significant differences in age, gender and educational level between homozygous *APOE* ε4 carriers and noncarriers (Table 2; data for separate age groups are reported in Table S3, S4, S5, S6, S7). However, there were some differences in cardiovascular risk factors. Homozygous *APOE* ε4 carriers had lower HDL cholesterol levels, and higher non-HDL cholesterol levels than noncarriers. On the other hand, homozygous *APOE* ε4 carriers had a lower frequency of elevated albuminuria than noncarriers (Table 2). There were no statistically significant differences for other cardiovascular risk factors.

**Table 2:** Demographic characteristics and major cardiovascular risk factors dependent on *APOE* ε4 genotype.

	Homozygous carrier	Heterozygous carrier	Noncarrier	*p*
**N**	101	1065	2689	N/A
**Gender, N (%)**				
Women	47 (47)	500 (47)	1312 (49)	0.56
Men	54 (53)	565 (53)	1377 (51)	
**Age, mean (SD), y**	54 (11)	54 (12)	55 (12)	0.07
**Age, N (%), y**				
35–44	23 (23)^a^	256 (24)^a^	568 (21)	
45–54	29 (29) a	331 (31)^a^	820 (31)	0.59
55–64	28 (28) a	247 (23)^a^	688 (25)	
65–74	17 (17) a	147 (16)^a^	466 (17)	
≥75	4 (4) a	57 (5)^a^	167 (6)	
**Educational level, N (%)**				
Primary school	8 (8)^a^	104 (10)	271 (10)	
Lower secondary education	37 (37)^a^	302 (28)	795 (30)	0.58
Higher secondary education	29 (29)^a^	289 (27)	725 (27)	
University	27 (27)^a^	370 (35)	898 (33)	
**Cardiovascular risk factors**				
Diabetes mellitus, N (%)	3 (3)	72 (7)	163 (6)	0.29
Current smoker, N (%)^d^	25 (25)	226 (21)	658 (25)	0.09
Body Mass Index, mean (SD), kg/m^2^	26 (3)	27 (4)	27 (4)	0.08
Systolic blood pressure, mean (SD), mmHg	125 (17)	126 (18)	126 (18)	0.87
Glucose, mean (SD), mmol/L^b^	4.8 (0.8)	4.9 (0.9)	4.9 (1.0)	0.79
Total cholesterol, mean (SD), mmol/L^c^	5.60 (1.10)	5.41 (1.07)	5.35 (1.05)	0.02
HDL cholesterol, mean (SD), mmol/L^c^	1.36 (0.41)	1.37 (0.38)	1.43 (0.38)	<0.001
Non-HDL cholesterol, mean (SD), mmol/L^c^	4.24 (0.99)	4.04 (1.03)	3.92 (1.02)	<0.001
Elevated albuminuria, N (%)^d^	6 (6)	151 (14)	402 (15)	0.04
**History, N (%)**				
Coronary heart disease	3 (3.0)	54 (5.1)	97 (3.6)	0.10
Cerebrovascular disease	1 (1.0)	11 (1.0)	21 (0.8)	e
**Current medication, N (%)** d				
Blood pressure lowering agents	17 (20)	239 (30)	615 (30)	0.15
Lipid lowering agents	16 (19)	182 (23)	345 (17)	0.001

N/A, not applicable; SD, standard deviation.

a Sum of the percentages is not equal to 100 due to rounding.

b Multiply by 18 to convert to mg/dL.

c Multiply by 39 to convert to mg/dL.

d Different total number due to missing data. For homozygous carriers, heterozygous carriers and noncarriers, data on smoking status were complete for 100, 1063, and 2671 persons, respectively; data on albuminuria were complete for 100, 1054, and 2667 persons, respectively; data on current medication were complete for 84, 792, and 2053 persons, respectively.

e Suppressed because of expected cell count of less than 1.

The difference in RFFT score between homozygous *APOE* ε4 carriers and noncarriers was also found in multiple regression analysis with adjustment for various covariables such as demographic characteristics and cardiovascular risk factors (Table 3). In the fully adjusted model, the RFFT score in homozygous *APOE* ε4 carriers was 5.24 points lower than in noncarriers (95% confidence interval, -9.41 to -1.07; *p* = 0.01). There was no interaction between *APOE* genotype and age.

**Table 3:** Multiple linear regression analysis of RFFT score on *APOE* ε4 genotype.

	Model 1^b^				Model 2^c^				Model 3^d^				Model 4^e^			
	B	SE(B)	β	*p*	B	SE(B)	β	*p*	B	SE(B)	β	*p*	B	SE(B)	β	*p*
***APOE*** ** ε4 genotype**																
Noncarrier	a				a				a				a			
Heterozygous carrier	2.15	0.94	0.04	0.02	1.05	0.81	0.02	0.19	1.02	0.75	0.02	0.17	1.00	0.77	0.02	0.19
Homozygous carrier	-4.66	2.64	-0.03	0.08	-5.83	2.26	-0.04	0.01	-4.61	2.09	-0.03	0.03	-5.24	2.13	-0.03	0.01
**Age, y**	-	-	-		-1.17	0.03	-0.52	<0.001	-0.92	0.03	-0.41	<0.001	-0.89	0.04	-0.40	<0.001
**Gender**																
Man	-	-	-		a				a				a			
Woman	-	-	-		-0.71	0.72	-0.13	0.33	0.38	0.67	0.007	0.57	-0.45	0.77	-0.009	0.56
**Educational level**																
Primary school	-	-	-		-	^-^	-		a				a			
Lower secondary education	-	-	-		-	^-^	-		6.66	1.23	0.12	<0.001	5.67	1.26	0.10	<0.001
Higher secondary education	-	-	-		-	-	-		14.89	1.28	0.25	<0.001	13.54	1.31	0.23	<0.001
University	-	-	-		^-^	-	-		25.22	1.25	0.46	<0.001	23.13	1.31	0.42	<0.001

RFFT, Ruff Figural Fluency Test; B, unstandardized coefficient; SE(B), standard error of B; β, standardized coefficient.

a Reference category

b Adjusted R^2^, 0.002; residual standard deviation, 26.

c Adjusted R^2^, 0.27; residual standard deviation, 22.

d Adjusted R^2^, 0.37; residual standard deviation, 21.

e Adjusted for the covariates in model 3 plus diabetes mellitus, current smoking status, body mass index, systolic blood pressure, glucose, HDL cholesterol, and elevated albuminuria. Adjusted R^2^, 0.38; residual standard deviation, 21.

If homozygous *APOE* ε4 carriers were compared with heterozygous *APOE* ε4 carriers, the mean RFFT score of homozygous carriers was 6.81 points lower (95% confidence interval, -12.15 to -1.47; *p* = 0.01). There were some differences in cardiovascular risk factors. The main difference was the higher prevalence of elevated albuminuria in heterozygous carriers (Table 2). After adjustment for the cardiovascular risk factors, the RFFT score in homozygous *APOE* ε4 carriers was 5.79 points lower than in heterozygous carriers (95% confidence interval, -10.71 to -5.12; *p* = 0.008).

There was no difference in RFFT score between heterozygous *APOE* ε4 carriers and noncarriers. In most age groups, heterozygous *APOE* ε4 carriers even had a higher RFFT score than noncarriers although the difference was small (Figure 1). Here also, there were small differences in cardiovascular risk factors between the groups (Table 2). However, adjustment for various covariables did not yield different results. In the fully adjusted model, RFFT scores in heterozygous *APOE* ε4 carriers and noncarriers were almost equal and the difference was not statistically significant (Table 3).

### Additional analyses

All additional analyses yielded essentially similar results.

#### Groningen Random Sample

This sample included 1,651 persons who performed the RFFT (69%). The *APOE* allele frequencies were equal to the frequencies in the total cohort. Unadjusted, the RFFT score in homozygous *APOE* ε4 carriers was 7.32 points lower than in noncarriers (95% confidence interval, -14.92 to 0.29; *p* = 0.06). In the fully adjusted model, the RFFT score in homozygous *APOE* ε4 carriers was 5.78 points lower than in noncarriers (95% confidence interval, -11.98 to 0.43; *p* = 0.07). There was no difference in RFFT score between heterozygous *APOE* ε4 carriers and noncarriers.

#### Exclusion persons of non-European descent

If the persons of non-European descent were exluded from the analysis, the unadjusted RFFT score in homozygous *APOE* ε4 carriers was 4.44 points lower than in noncarriers (95% confidence interval, -9.75 to 0.87; *p* = 0.10). In the fully adjusted model, the RFFT score in homozygous *APOE* ε4 carriers was 5.11 points lower than in noncarriers (95% confidence interval, -9.37 to -0.85; *p* = 0.02). There was no difference in RFFT score between heterozygous *APOE* ε4 carriers and noncarriers.

#### Exclusion of APOE ε2/ε4 carriers

After exclusion of the *APOE* ε2/ε4 carriers, the unadjusted RFFT in homozygous *APOE* ε4 carriers was 6.97 points lower than in heterozygous *APOE* ε4 carriers (95% confidence interval, -12.32 to -1.62; *p* = 0.01), and 4.66 points lower than in noncarriers (95% confidence interval, -9.84 to 0.51; *p* = 0.08). In the fully adjusted models, the RFFT score in homozygous *APOE* ε4 carriers was 5.72 points lower than in heterozygous *APOE* ε4 carriers (95% confidence interval, -9.99 to -1.45; *p* = 0.009), and 5.23 points lower than in noncarriers (95% confidence interval, -9.67 to -1.39; *p* = 0.009).

In addition, the unadjusted RFFT score in heterozygous *APOE* ε4 carriers was 2.31 points higher than in noncarriers (95% confidence interval, 0.41 to 4.22; *p* = 0.02). In the fully adjusted model, heterozygous *APOE* ε4 carriers and noncarriers had similar RFFT scores (mean difference, 0.96 points; 95% confidence interval, -0.58 to 2.51; *p* = 0.22).

#### Exclusion of all APOE ε2 carriers

After exclusion of all *APOE* ε2 carriers, the unadjusted RFFT score in homozygous *APOE* ε4 carriers was 4.98 points lower than in noncarriers (95% confidence interval, -10.17 to 0.21; *p* = 0.06). In the fully adjusted model, the RFFT score in homozygous *APOE* ε4 carriers was 7.91 points lower than in noncarriers (95% confidence interval, -12.46 to -3.36; *p* = 0.001). Similar differences were found if homozygous *APOE* ε4 carriers were compared with heterozygous *APOE* ε4 carriers. There was no difference in RFFT score between heterozygous *APOE* ε4 carriers and noncarriers.

## Discussion

In this study, *APOE* ε4 homozygous carriers had worse cognitive function than *APOE* ε4 heterozygous carriers and noncarriers. This was not only found in older persons but across all ages beginning at the age of 35 years. Remarkably, there was no difference in cognitive function between *APOE* ε4 heterozygous carriers and noncarriers. Thus, the effect of the *APOE* ε4 allele on cognitive function was not dose-dependent in this study.

There are several studies on the association of *APOE* genotype with cognitive function in young as well as older persons, but to our knowledge, only three studies had sample sizes that were large enough to draw reliable conclusions [3]–[6]. In one study, that was part of a community survey and included persons who were 20 to 64 years of age [3], [6], *APOE* genotype was not associated with cognitive function. Surprisingly, this lack of association was not only found in the young age groups but also in the old age group. The authors suggested that the effects of *APOE* genotype only occur above the age of 65 years but this explanation does not seem plausible and is not supported by the findings of the two other studies. The two other studies included persons aged 21 years or older but were performed in selected subjects as one study sample essentially consisted of one extended family [4],and the other study sample was strongly enriched for *APOE* ε4 carriers [5]. In these studies, cognitive function of *APOE* ε4 carriers and noncarriers was comparable when they were aged 40 years or younger. The difference in cognitive function between *APOE* ε4 carriers and noncarriers became first detectable between the ages of 40 and 55 years and then gradually increased with increasing age. In contrast, in our community-based study, the difference in cognitive function between *APOE* ε4 homozygous carriers and noncarriers was found in young as well as older persons, and the magnitude of the difference in cognitive performance was similar across all ages. Possibly, the differences in findings between our study and the other two positive studies can be explained by the difference in cognitive domains that were tested.

In the other two positive studies, *APOE* ε4 carriers were different from noncarriers if cognitive function was measured with a verbal learning test [4], [5]. In contrast to verbal learning, (figural) fluency is dependent on cognitive abilities such as reaction time, pattern recognition and problem solving. Verbal learning and fluency have also been related to different brain regions. Whereas verbal learning is usually associated with the hippocampus and medial temporal lobe, fluency is usually associated with the prefrontal lobes [21], [22]. However, genetic effects may vary between brain regions [23]. Therefore, the difference in association of *APOE* genotype with performance on verbal learning and fluency tests may be due to a different effect of ApoE on the medial temporal and prefrontal lobes.

The effect of ApoE on cognitive function is commonly ascribed to its role in the accumulation of amyloid-β in the brain [2]. However, *APOE* ε4 carriers and noncarriers do not clearly differ in this aspect until the age of 50 to 59 years [24]. As a consequence, our findings can not be explained by differences in amyloid-β accumulation because in our study, the difference in cognitive function between *APOE* ε4 carriers and noncarriers was already present before the age of 50 years. An alternative explanation for the difference in cognitive function at a relatively young age can be sought in the role of ApoE in the growth and development of the brain. ApoE may, for example, interfere with the binding of reelin to ApoE receptors, a pathway that is crucial for neuronal migration and dendritic spine development [2]. This mechanism may not only affect the development of gray matter but also the development of white matter. White matter is essential for brain connectivity and integrates gray matter regions into neural networks [25]. More importantly, white matter lesions are in general more related to impairment of cognitive abilities that are essential for fluency than to memory dysfunction [26], [27]. Interestingly, it was recently found that *APOE* genotype dependent differences in white matter structure are already present in young adulthood and do not undergo significant differential changes with age [28].

Although there was a difference between *APOE* ε4 carriers and noncarriers in performance on a verbal learning test in the previous studies, no differences were found on tests of other (nonmemory) cognitive domains [4], [5]. Some of these tests, such as the Trail Making Test and the Stroop Color-Word Test [29], [30], are generally seen as a measure of executive function and thus reflect similar cognitive abilities as the RFFT, that was used in our study. Therefore, it can be questioned why no association of *APOE* genotype with performance on these tests was found in the previous studies. In fact, it does not seem plausible that a genotype that is associated with an increased risk of Alzheimer's disease is not associated with decreased performance in other cognitive domains as by definition, Alzheimer's disease affects more than one cognitive domain [31]. A possible explanation for the divergent finding on the association of *APOE* genotype with performance on executive function tests may be that the RFFT with its score range of 0 to 175 points is more sensitive to differences in performance than the tests that were use in the other studies.

Another important difference between the studies was the effect of *APOE* ε4 heterozygosity. In the previous studies, *APOE* ε4 heterozygous carriers had better cognitive function than *APOE* ε4 homozygous carriers but worse cognitive function than noncarriers. In our study, the cognitive function of *APOE* ε4 heterozygous carriers was similar to that of the noncarriers. If it is assumed that ApoE plays a role in the growth and development of certain brain regions, this would mean that growth and development of these regions is only affected if persons have two *APOE* ε4 alleles and not if persons have only one *APOE* ε4 allele.

Homozygous *APOE* ε4 carriers only express the ApoE4 isoform of apolipoprotein E but heterozygous *APOE* ε4 carriers also express the ApoE2 or ApoE3 isoform. These other isoforms of apoliprotein E are more effective in regulating brain lipid metabolism and synaptic functions. ApoE3, for example, is more effective than ApoE4 in transporting cholesterol in the brain and in maintaining neurons [2]. Interestingly, it was found in brain imaging studies that homozygous *APOE* ε4 carriers have considerably more white matter lesions than heterozygous *APOE* ε4 carriers and noncarriers whereas the load of white matter lesions in heterozygous *APOE* ε4 carriers and noncarriers was comparable [32], [33]. Thus, the presence of one other ApoE isoform may be sufficient for attaining normal development and maintenance of the brain.

There are some methodological aspects to our study that need to be acknowledged. First, the cross-sectional design may be considered as a limitation. In general, one should be cautious about inferring a causal relationship from a cross-sectional study. However, *APOE* genotype is a proven risk factor for Alzheimer's disease and cognitive impairment in old age [1]. Consequentially, it is unlikely that we observed a difference in cognitive performance between homozygous *APOE* ε4 carriers and noncarriers when in truth there was none. Second, it should be mentioned that we used only one test of cognitive function. Even so, the RFFT score is the outcome of many different cognitive abilities. Moreover, the RFFT is a sensitive cognitive test so that it was possible to measure differences in cognitive performance in relatively young persons. Additionally, in contrast to many other tests, the RFFT does not seem to exhibit a strong floor or ceiling effect as it has a wide range of scores. Third, due to the design of the PREVEND study, the study sample included a relatively high proportion of persons with elevated albuminuria and hence, with increased cardiovascular risk. However, similar results were found when the analyses were repeated in a random sample from the general population. Finally, we did not conduct a replication analysis because no independent replication cohort was available. Therefore, our findings should be interpreted with some caution.

In conclusion, homozygous *APOE* ε4 carriers aged 35 years and older had worse cognitive function than heterozygous *APOE* ε4 carriers and noncarriers of comparable age. The difference in cognitive performance was not dependent on age and its magnitude was similar for all ages.

## Supporting Information

Table S1 **Ethnicity of the study population.**
(DOC)

Table S2 **Distribution of** ***APOE*** **ε4 genotypes dependent on ethnicity.**
(DOC)

Table S3 **Demographic characteristics and major cardiovascular risk factors dependent on** ***APOE*** **ε4 genotype: age group 35 to 44 years.**
(DOC)

Table S4 **Demographic characteristics and major cardiovascular risk factors dependent on** ***APOE*** **ε4 genotype: age group 45 to 54 years.**
(DOC)

Table S5 **Demographic characteristics and major cardiovascular risk factors dependent on** ***APOE*** **ε4 genotype: age group 55 to 64 years.**
(DOC)

Table S6 **Demographic characteristics and major cardiovascular risk factors dependent on** ***APOE*** **ε4 genotype: age group 65 to 74 years.**
(DOC)

Table S7 **Demographic characteristics and major cardiovascular risk factors dependent on** ***APOE*** **ε4 genotype: age group 75 years or older.**
(DOC)
