# Supplementary material for: The Association of APOE Genotype with Cognitive Function in Persons Aged 35 Years or Older
Source: PLoS One. 2011 Nov 14;6(11):e27415. doi: 10.1371/journal.pone.0027415 (PMC3215744; doi:10.1371/journal.pone.0027415)
Supplement: Table S1 — Ethnicity of the study population. (DOC) [file pone.0027415.s001.doc]

**Table S1. Ethnicity of the study population.**

| **Ethnicity** | **Men^a^** | | **Women^a^** | | **All^a^** | |
| --- | --- | --- | --- | --- | --- | --- |
|  | **N** | **%** | **N** | **%** | **N** | **%** |
| European | 2067 | 96 | 1896 | 96 | 3963 | 96 |
| African | 15 | 1 | 15 | 1 | 30 | 1 |
| Asian | 38 | 2 | 35 | 2 | 73 | 2 |
| Other | 21 | 1 | 14 | 1 | 35 | 1 |
| Missing | 16 | 1 | 18 | 1 | 34 | 1 |
| All | 2157 | 100.0 | 1978 | 100.0 | 4135 | 100.0 |

^a^ Sum of the percentages is not equal to 100 due to rounding.
