# Supplementary material for: The Association of APOE Genotype with Cognitive Function in Persons Aged 35 Years or Older
Source: PLoS One. 2011 Nov 14;6(11):e27415. doi: 10.1371/journal.pone.0027415 (PMC3215744; doi:10.1371/journal.pone.0027415)
Supplement: Table S2 — Distribution of APOE ε4 genotypes dependent on ethnicity. (DOC) [file pone.0027415.s002.doc]

**Table S2. Distribution of *APOE* ε4 genotypes dependent on ethnicity.**

|  | **Ethnicity^b^** | | | | | | | | | | | |
| --- | --- | --- | --- | --- | --- | --- | --- | --- | --- | --- | --- | --- |
|  | **European** | | **African** | | **Asian** | | **Other** | | **Missing** | | **All** | |
| ***APOE* ε4 genotype^a^** | **N** | **%** | **N** | **%** | **N** | **%** | **N** | **%** | **N** | **%** | **N** | **%** |
| Noncarrier | 2574 | 70 | 14 | 52 | 55 | 81 | 25 | 81 | 21 | 68 | 2689 | 70 |
| Heterozygous | 1028 | 28 | 10 | 37 | 12 | 18 | 6 | 19 | 9 | 29 | 1065 | 28 |
| Homozygous | 96 | 3 | 3 | 11 | 1 | 2 | 0 | 0 | 1 | 3 | 101 | 3 |
| All | 3698 | 100 | 27 | 100 | 68 | 100 | 31 | 100 | 31 | 100 | 3855 | 100 |

^a^ *APOE* genotype was determined in 3855 persons (93% of the total study population).

^b^ χ^2^ test was not performed because of expected cell counts of less than one.
