# Supplementary material for: The Association of APOE Genotype with Cognitive Function in Persons Aged 35 Years or Older
Source: PLoS One. 2011 Nov 14;6(11):e27415. doi: 10.1371/journal.pone.0027415 (PMC3215744; doi:10.1371/journal.pone.0027415)
Supplement: Table S3 — Demographic characteristics and major cardiovascular risk factors dependent on APOE ε4 genotype: age group 35 to 44 years. (DOC) [file pone.0027415.s003.doc]

**Table S3. Demographic characteristics and major cardiovascular risk factors dependent on *APOE* ε4 genotype: age group 35 to 44 years.**

|  | **Homozygous carrier** | **Heterozygous carrier** | **Noncarrier** | ***p*** |
| --- | --- | --- | --- | --- |
| **N^a^** | 23 | 256 | 568 | N/A |
| **Gender, N (%)** |  |  |  |  |
| Women | 11 (48) | 125 (49) | 288 (51) | 0.86 |
| Men | 12 (52) | 131 (51) | 280 (49) |  |
| **Age, mean (SD), y** | 40 (3) | 40 (3) | 40 (3) | 0.34 |
| **Educational level, N (%)** |  |  |  |  |
| Primary school | 0 (0) | 2 (1) | 18 (3)^b^ |  |
| Lower secondary education | 3 (13) | 42 (16) | 86 (15) ^b^ | c |
| Higher secondary education | 13 (57) | 91 (36) | 207 (36) ^b^ |  |
| University | 7 (30) | 121 (47) | 257 (45) ^b^ |  |
| **Cardiovascular risk factors** |  |  |  |  |
| Diabetes mellitus, N (%) | 0 (0) | 2 (1) | 4 (1) | c |
| Current smoker, N (%)^d^ | 8 (35) | 45 (18) | 145 (26) | 0.02 |
| Body Mass Index, mean (SD), kg/m^2^ | 25 (3) | 26 (4) | 25 (4) | 0.47 |
| Systolic blood pressure, mean (SD), mmHg | 115 (11) | 118 (13) | 117 (12) | 0.23 |
| Glucose, mean (SD), mmol/L^e^ | 4.5 (0.6) | 4.6 (0.5) | 4.5 (0.6) | 0.73 |
| Total cholesterol, mean (SD), mmol/L^f^ | 5.22 (0.71) | 5.23 (0.95) | 5.08 (0.93) | 0.08 |
| HDL cholesterol, mean (SD), mmol/L^f^ | 1.27 (0.36) | 1.37 (0.34) | 1.42 (0.35) | 0.03 |
| Non-HDL cholesterol, mean (SD), mmol/L^f^ | 3.95 (0.70) | 3.86 (0.97) | 3.66 (0.96) | 0.01 |
| Elevated albuminuria, N (%)^d^ | 1 (4) | 16 (6) | 30 (5) | 0.80 |
| **History, N (%)** |  |  |  |  |
| Coronary heart disease | 1 (4) | 0 (0) | 3 (1) | c |
| Cerebrovascular disease | 0 (0) | 0 (0) | 0 (0) | c |
| **Current medication, N (%)^d^** |  |  |  |  |
| Blood pressure lowering agents | 2 (13) | 5 (3) | 18 (5) | c |
| Lipid lowering agents | 3 (19) | 8 (5) | 6 (2) | c |

N/A, not applicable; SD, standard deviation.

^a^ In this age group, *APOE* genotype was determined in 847 persons (92%).

^b^ Sum of the percentages is not equal to 100 due to rounding.

^c^ Suppressed because of expected cell count of less than one.

^d^ Different total number due to missing data. For homozygous carriers, heterozygous carriers and noncarriers, data on smoking status were complete for 23, 256, and 565 persons, respectively; data on albuminuria were complete for 23, 252, and 567 persons, respectively; data on current medication were complete for 16, 152, and 335 persons, respectively.

^e^ Multiply by 18 to convert to mg/dL.

^f^ Multiply by 39 to convert to mg/dL.
