# Supplementary material for: The Association of APOE Genotype with Cognitive Function in Persons Aged 35 Years or Older
Source: PLoS One. 2011 Nov 14;6(11):e27415. doi: 10.1371/journal.pone.0027415 (PMC3215744; doi:10.1371/journal.pone.0027415)
Supplement: Table S4 — Demographic characteristics and major cardiovascular risk factors dependent on APOE ε4 genotype: age group 45 to 54 years. (DOC) [file pone.0027415.s004.doc]

**Table S4. Demographic characteristics and major cardiovascular risk factors dependent on *APOE* ε4 genotype: age group 45 to 54 years.**

|  | **Homozygous carrier** | **Heterozygous carrier** | **Noncarrier** | ***p*** |
| --- | --- | --- | --- | --- |
| **N^a^** | 29 | 331 | 820 | N/A |
| **Gender, N (%)** |  |  |  |  |
| Women | 13 (45) | 164 (49) | 435 (53) | 0.42 |
| Men | 16 (55) | 167 (51) | 385 (47) |  |
| **Age, mean (SD), y** | 50 (3) | 50 (3) | 50 (3) | 0.71 |
| **Educational level, N (%)** |  |  |  |  |
| Primary school | 3 (10) | 22 (7)^b^ | 45 (6) ^b^ |  |
| Lower secondary education | 12 (41) | 73 (22) ^b^ | 222 (27) ^b^ | 0.13 |
| Higher secondary education | 4 (14) | 101 (31) ^b^ | 220 (27) ^b^ |  |
| University | 10 (35) | 135 (41) ^b^ | 333 (41) ^b^ |  |
| **Cardiovascular risk factors** |  |  |  |  |
| Diabetes mellitus, N (%) | 1 (3) | 14 (4) | 21 (3) | c |
| Current smoker, N (%)^d^ | 6 (21) | 91 (28) | 256 (31) | 0.23 |
| Body Mass Index, mean (SD), kg/m^2^ | 26 (3) | 26 (4) | 26 (5) | 0.96 |
| Systolic blood pressure, mean (SD), mmHg | 123 (16) | 122 (16) | 122 (15) | 0.41 |
| Glucose, mean (SD), mmol/L^e^ | 5.0 (0.8) | 4.8 (0.9) | 4.7 (0.7) | 0.13 |
| Total cholesterol, mean (SD), mmol/L^f^ | 5.95 (1.07) | 5.54 (1.04) | 5.41 (1.04) | 0.006 |
| HDL cholesterol, mean (SD), mmol/L^f^ | 1.41 (0.36) | 1.39 (0.36) | 1.44 (0.38) | 0.13 |
| Non-HDL cholesterol, mean (SD), mmol/L^f^ | 4.54 (1.08) | 4.15 (1.03) | 3.97 (1.05) | 0.001 |
| Elevated albuminuria, N (%)^d^ | 1 (3) | 42 (13) | 83 (10) | 0.19 |
| **History, N (%)** |  |  |  |  |
| Coronary heart disease | 0 (0) | 13 (4) | 15 (2) | c |
| Cerebrovascular disease | 0 (0) | 4 (1) | 3 (0) | c |
| **Current medication, N (%)^d^** |  |  |  |  |
| Blood pressure lowering agents | 5 (20) | 45 (19) | 110 (18) | 0.88 |
| Lipid lowering agents | 3 (12) | 39 (17) | 66 (11) | 0.06 |

N/A, not applicable; SD, standard deviation.

^a^ In this age group, *APOE* genotype was determined in 1180 persons (92%).

^b^ Sum of the percentages is not equal to 100 due to rounding.

^c^ Suppressed because of expected cell count of less than one.

^d^ Different total number due to missing data. For homozygous carriers, heterozygous carriers and noncarriers, data on smoking status were complete for 29, 331, and 815 persons, respectively; data on albuminuria were complete for 29, 328, and 813 persons, respectively; data on current medication were complete for 25, 235, and 617 persons, respectively.

^e^ Multiply by 18 to convert to mg/dL.

^f^ Multiply by 39 to convert to mg/dL.
