# Supplementary material for: The Association of APOE Genotype with Cognitive Function in Persons Aged 35 Years or Older
Source: PLoS One. 2011 Nov 14;6(11):e27415. doi: 10.1371/journal.pone.0027415 (PMC3215744; doi:10.1371/journal.pone.0027415)
Supplement: Table S5 — Demographic characteristics and major cardiovascular risk factors dependent on APOE ε4 genotype: age group 55 to 64 years. (DOC) [file pone.0027415.s005.doc]

**Table S5. Demographic characteristics and major cardiovascular risk factors dependent on *APOE* ε4 genotype: age group 55 to 64 years.**

|  | **Homozygous carrier** | **Heterozygous carrier** | **Noncarrier** | ***p*** |
| --- | --- | --- | --- | --- |
| **N^a^** | 28 | 247 | 668 | N/A |
| **Gender, N (%)** |  |  |  |  |
| Women | 17 (61) | 120 (49) | 338 (51) | 0.47 |
| Men | 11 (39) | 127 (51) | 330 (49) |  |
| **Age, mean (SD), y** | 58 (3) | 59 (3) | 59 (3) | 0.58 |
| **Educational level, N (%)** |  |  |  |  |
| Primary school | 3 (11) ^b^ | 31 (13) ^b^ | 74 (11) |  |
| Lower secondary education | 10 (36) ^b^ | 88 (36) ^b^ | 228 (34) | 0.98 |
| Higher secondary education | 8 (29) ^b^ | 59 (24) ^b^ | 168 (25) |  |
| University | 7 (25) ^b^ | 69 (28) ^b^ | 198 (30) |  |
| **Cardiovascular risk factors** |  |  |  |  |
| Diabetes mellitus, N (%) | 0 (0) | 24 (10) | 56 (8) | 0.21 |
| Current smoker, N (%)^d^ | 7 (26) | 53 (22) | 148 (22) | 0.87 |
| Body Mass Index, mean (SD), kg/m^2^ | 26 (3) | 27 (5) | 28 (5) | 0.07 |
| Systolic blood pressure, mean (SD), mmHg | 127 (18) | 126 (17) | 127 (17) | 0.80 |
| Glucose, mean (SD), mmol/L^e^ | 5.0 (0.7) | 5.1 (1.1) | 5.1 (1.2) | 0.69 |
| Total cholesterol, mean (SD), mmol/L^f^ | 5.91 (0.98) | 5.48 (1.16) | 5.60 (1.06) | 0.09 |
| HDL cholesterol, mean (SD), mmol/L^f^ | 1.39 (0.44) | 1.39 (0.42) | 1.47 (0.42) | 0.03 |
| Non-HDL cholesterol, mean (SD), mmol/L^f^ | 4.52 (0.89) | 4.09 (1.08) | 4.14 (0.99) | 0.10 |
| Elevated albuminuria, N (%)^d^ | 0 (0) | 38 (16) | 98 (15) | 0.09 |
| **History, N (%)** |  |  |  |  |
| Coronary heart disease | 1 (4) | 19 (8) | 26 (4) | 0.06 |
| Cerebrovascular disease | 0 (0) | 0 (0) | 7 (1) | c |
| **Current medication, N (%)^d^** |  |  |  |  |
| Blood pressure lowering agents | 6 (25) | 82 (40) | 178 (33) | 0.11 |
| Lipid lowering agents | 3 (13) | 58 (29) | 111 (21) | 0.04 |

N/A, not applicable; SD, standard deviation.

^a^ In this age group, *APOE* genotype was determined in 943 persons (94%).

^b^ Sum of the percentages is not equal to 100 due to rounding.

^c^ Suppressed because of expected cell count of less than one.

^d^ Different total number due to missing data. For homozygous carriers, heterozygous carriers and noncarriers, data on smoking status were complete for 27, 246, and 665 persons, respectively; data on albuminuria were complete for 27, 244, and 662 persons, respectively; data on current medication were complete for 24, 203, and 536 persons, respectively.

^e^ Multiply by 18 to convert to mg/dL.

^f^ Multiply by 39 to convert to mg/dL.
