# Supplementary material for: The Association of APOE Genotype with Cognitive Function in Persons Aged 35 Years or Older
Source: PLoS One. 2011 Nov 14;6(11):e27415. doi: 10.1371/journal.pone.0027415 (PMC3215744; doi:10.1371/journal.pone.0027415)
Supplement: Table S6 — Demographic characteristics and major cardiovascular risk factors dependent on APOE ε4 genotype: age group 65 to 74 years. (DOC) [file pone.0027415.s006.doc]

**Table S6. Demographic characteristics and major cardiovascular risk factors dependent on *APOE* ε4 genotype: age group 65 to 74 years.**

|  | **Homozygous carrier** | **Heterozygous carrier** | **Noncarrier** | ***p*** |
| --- | --- | --- | --- | --- |
| **N^a^** | 17 | 174 | 466 | N/A |
| **Gender, N (%)** |  |  |  |  |
| Women | 6 (35) | 68 (39) | 194 (42) | 0.76 |
| Men | 11 (65) | 106 (41) | 272 (58) |  |
| **Age, mean (SD), y** | 68 (3) | 69 (3) | 69 (3) | 0.44 |
| **Educational level, N (%)** |  |  |  |  |
| Primary school | 1 (6)^b^ | 37 (21) ^b^ | 93 (20) |  |
| Lower secondary education | 10 (59) ^b^ | 70 (40) ^b^ | 200 (43) | c |
| Higher secondary education | 4 (24) ^b^ | 28 (16) ^b^ | 92 (20) |  |
| University | 2 (12) ^b^ | 39 (22) ^b^ | 81 (17) |  |
| **Cardiovascular risk factors** |  |  |  |  |
| Diabetes mellitus, N (%) | 2 (12) | 26 (15) | 47 (10) | 0.23 |
| Current smoker, N (%)^e^ | 4 (24) | 31 (18) | 91 (20) | 0.79 |
| Body Mass Index, mean (SD), kg/m^2^ | 28 (3) | 28 (4) | 28 (4) | 0.71 |
| Systolic blood pressure, mean (SD), mmHg | 131 (14) | 137 (19) | 136 (19) | 0.45 |
| Glucose, mean (SD), mmol/L^f^ | 5.0 (1.1) | 5.2 (1.1) | 5.1 (1.1) | 0.40 |
| Total cholesterol, mean (SD), mmol/L^g^ | 5.06 (1.51) | 5.31 (1.14) | 5.24 (1.06) | 0.60 |
| HDL cholesterol, mean (SD), mmol/L^g^ | 1.37 (0.53) | 1.31 (0.38) | 1.39 (0.38) | 0.05 |
| Non-HDL cholesterol, mean (SD), mmol/L^g^ | 3.70 (1.14) | 4.00 (1.03) | 3.84 (1.01) | 0.17 |
| Elevated albuminuria, N (%)^e^ | 3 (18) | 35 (20) | 127 (28) | 0.12 |
| **History, N (%)** |  |  |  |  |
| Coronary heart disease | 1 (6) | 19 (11) | 36 (8) | 0.40 |
| Cerebrovascular disease | 0 (0) | 6 (3) | 8 (2) | d |
| **Current medication, N (%)^e^** |  |  |  |  |
| Blood pressure lowering agents | 3 (20) | 73 (50) | 220 (53) | 0.04 |
| Lipid lowering agents | 6 (40) | 56 (38) | 125 (30) | 0.18 |

N/A, not applicable; SD, standard deviation.

^a^ In this age group, *APOE* genotype was determined in 657 persons (95%).

^b^ Sum of the percentages is not equal to 100 due to rounding.

^c^ Suppressed because more than one fifth of cells have expected cell counts less than five.

^d^ Suppressed because of expected cell count of less than one.

^e^ Different total number due to missing data. For homozygous carriers, heterozygous carriers and noncarriers, data on smoking status were complete for 17, 173, and 460 persons, respectively; data on albuminuria were complete for 17, 173, and 459 persons, respectively; data on current medication were complete for 15, 147, and 414 persons, respectively.

^f^ Multiply by 18 to convert to mg/dL.

^g^ Multiply by 39 to convert to mg/dL.
