# Supplementary material for: The Association of APOE Genotype with Cognitive Function in Persons Aged 35 Years or Older
Source: PLoS One. 2011 Nov 14;6(11):e27415. doi: 10.1371/journal.pone.0027415 (PMC3215744; doi:10.1371/journal.pone.0027415)
Supplement: Table S7 — Demographic characteristics and major cardiovascular risk factors dependent on APOE ε4 genotype: age group 75 years or older. (DOC) [file pone.0027415.s007.doc]

**Table S7. Demographic characteristics and major cardiovascular risk factors dependent on *APOE* ε4 genotype: age group 75 years or older.**

|  | **Homozygous carrier** | **Heterozygous carrier** | **Noncarrier** | ***p*** |
| --- | --- | --- | --- | --- |
| **N^a^** | 4 | 57 | 167 | N/A |
| **Gender, N (%)** |  |  |  |  |
| Women | 0 (0) | 23 (40) | 57 (34) | c |
| Men | 4 (100) | 34 (60) | 110 (66) |  |
| **Age, mean (SD), y** | 76 (75, 78) | 76 (75, 78) | 78 (76, 79) | 0.04 |
| **Educational level, N (%)** |  |  |  |  |
| Primary school | 1 (25) | 12 (21)^b^ | 41 (25) |  |
| Lower secondary education | 2 (50) | 29 (51) ^b^ | 59 (35) | d |
| Higher secondary education | 0 (0) | 10 (18) ^b^ | 38 (23) |  |
| University | 1 (25) | 6 (11) ^b^ | 29 (17) |  |
| **Cardiovascular risk factors** |  |  |  |  |
| Diabetes mellitus, N (%) | 0 (0) | 6 (11) | 35 (21) | d |
| Current smoker, N (%) | 0 (0) | 6 (11) | 18 (11) | d |
| Body Mass Index, mean (SD), kg/m^2^ | 24 (2) | 28 (4) | 28 (4) | 0.18 |
| Systolic blood pressure, mean (SD), mmHg | 154 (14) | 143 (21) | 144 (19) | 0.57 |
| Glucose, mean (SD), mmol/L^f^ | 4.2 (1.2) | 4.9 (0.9) | 5.3 (1.4) | 0.02 |
| Total cholesterol, mean (SD), mmol/L^g^ | 5.36 (55) | 5.43 (1.11) | 5.19 (1.07) | 0.34 |
| HDL cholesterol, mean (SD), mmol/L^g^ | 1.18 (21) | 1.37 (0.42) | 1.30 (0.35) | 0.37 |
| Non-HDL cholesterol, mean (SD), mmol/L^g^ | 4.18 (59) | 4.06 (1.03) | 3.89 (1.01) | 0.36 |
| Elevated albuminuria, N (%) | 1 (25) | 20 (35) | 64 (39) | c |
| **History, N (%)** |  |  |  |  |
| Coronary heart disease | 0 (0) | 3 (5) | 17 (10) | d |
| Cerebrovascular disease | 1 (25) | 1 (2) | 3 (2) | d |
| **Current medication, N (%)^e^** |  |  |  |  |
| Blood pressure lowering agents | 1 (25) | 34 (62) | 89 (59) | c |
| Lipid lowering agents | 1 (25) | 21 (38) | 37 (25) | c |

N/A, not applicable; SD, standard deviation.

^a^ In this age group, *APOE* genotype was determined in 228 persons (93%).

^b^ Sum of the percentages is not equal to 100 due to rounding.

^c^ Suppressed because more than one fifth of cells have expected cell counts less than five.

^d^ Suppressed because of expected cell count of less than one.

^e^ Different total number due to missing data. For homozygous carriers, heterozygous carriers and noncarriers, data on on current medication were complete for 4, 55, and 151 persons, respectively.

^f^ Multiply by 18 to convert to mg/dL.

^g^ Multiply by 39 to convert to mg/dL
